# The Role of Genetics, Epigenetics, and the Environment in ASD: A Mini Review

**DOI:** 10.3390/epigenomes6020015

**Published:** 2022-06-19

**Authors:** Asim A. Khogeer, Iman S. AboMansour, Dia A. Mohammed

**Affiliations:** 1 Research Department, The Strategic Planning Administration, General Directorate of Health Affairs of Makkah Region, Ministry of Health, Makkah 24382, Saudi Arabia; 2 Medical Genetics Unit, Maternity & Children Hospital, Makkah Healthcare Cluster, Ministry of Health, Makkah 24382, Saudi Arabia; dimohammed@moh.gov.sa; 3 Scientific Council, Molecular Research and Training Center, iGene, Jeddah 3925, Saudi Arabia; 4 Department of Medical Genetics, Faculty of Medicine, Umm Al-Qura University, Makkah 24382, Saudi Arabia; isabumansour@uqu.edu.sa; 5 Neurogenetic Section, Department of Pediatrics, King Faisal Specialist Hospital and Research Center, Jeddah 2865, Saudi Arabia

**Keywords:** autism spectrum disorder, genetics, molecular mechanisms, epigenetics, DNA methylation, rare variants, common variants

## Abstract

According to recent findings, variances in autism spectrum disorder (ASD) risk factors might be determined by several factors, including molecular genetic variants. Accumulated evidence has also revealed the important role of biological and chemical pathways in ASD aetiology. In this paper, we assess several reviews with regard to their quality of evidence and provide a brief outline of the presumed mechanisms of the genetic, epigenetic, and environmental risk factors of ASD. We also review some of the critical literature, which supports the basis of each factor in the underlying and specific risk patterns of ASD. Finally, we consider some of the implications of recent research regarding potential molecular targets for future investigations.

## 1. Introduction

Autistic disorder—or, more broadly, autism spectrum disorders (ASD)—is a lifelong syndrome with a childhood inception, characterised by challenges in social interaction and communication, the presence of stereotype rigidity, and ritualistic/repetitive patterns of behaviour [1]. ASD syndrome has been described by many researchers since it was first observed in 1943 by Kanner, who described 11 cases of children, mostly boys, with severe social and variable language dysfunction [2]. The global prevalence of autism is around one percent, with a male preponderance ratio of four to one [3]. Approximately 50 percent of patients with ASD present with intellectual disabilities (ID), and comorbidity with neurodevelopmental and psychiatric conditions is common [3,4]. These psychiatric and medical conditions may include depression, anxiety, attention deficit hyperactivity disorder (ADHD), sleep illnesses, and gastrointestinal symptoms [5,6,7]. In addition, more than 35 percent of autistic individuals suffer from epilepsy, and epileptic EEG abnormalities can often be found in patients even without the occurrence of seizures [8,9]. However, the main diagnostic principle of autistic disorder is based upon the consensus opinion of expert clinicians, albeit with the consideration of the modified criteria under the new ASD heading [10].

**Figure 1:**
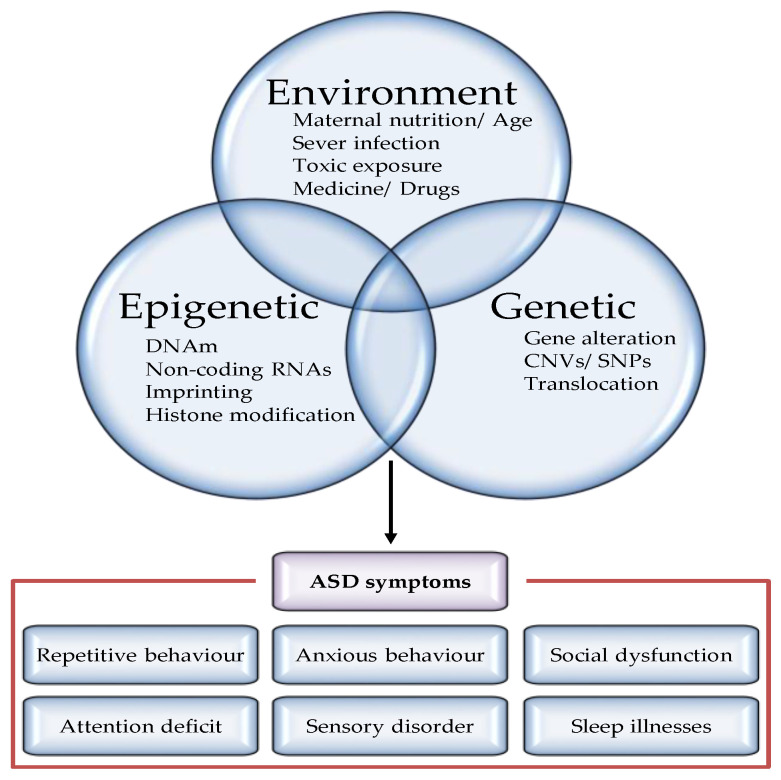
Factors regulating the pathogenesis of autism spectrum disorders. Although definitive etiology and pathogenesis underlying ASD have not yet been identified, accumulated study has recognized various risk factors, including nature (genes or epigenes) and nurture (environment) factors. Both genetic and epigenetic factors modulate the penetrance of risk genes, resulting in a highly heterogeneous disease phenotype for similar pathogenic variants. Examples of genetic modulators include CNV and mutations. Examples of epigenetic modifiers include methylation, microRNAs (miRNAs), and chromatin remodelling. Furthermore, majority of the environmental factors leading to epigenetic changes on chromatin cause increased ASD risk.

Incontrovertible evidence found in twin studies has proven that genetic factors contribute to susceptibility to this disorder [11,12]. The concordance rate of autism is up to 30 percent in dizygotic twins (DZ), 70–90 percent in monozygotic twins (MZ), and 3–19 percent in siblings in general [13,14,15,16,17]. The higher disorder cooccurrence in MZ twins (identical in their genetic material) compared to DZ twins (sharing about 50 percent of their genetic material like nontwin siblings) seems to support a genetic aetiology [10]. In terms of aetiology, for example, family studies comparing the frequency of autism between the first-degree relatives of affected individuals and the wider population should consider the involvement of genetics in the disease. Another area of evidence that supports the genetic aetiology of ASD can be found in studies on rare genetic syndromes with comorbid autism diagnoses. Findings in these studies have shown that sharing the same in utero environment has a more impactful role than genetics on siblings; however, the mechanism underlying this notion is still unclear [10,14,18]. Moreover, it has been shown that molecular alterations can contribute to ASD aetiology, including altered genetic and epigenetic regulation. Epigenetic mechanisms provide new and critical ways to examine risk estimates for neurodevelopmental disorders (NDDs) beyond genetic risk alone. These mechanisms may include DNA methylation (DNAm), histone modification, and ATP-dependent chromatin remodelling; however, the latter system also modulates the transcriptome and splicing processes, thus impacting transcription initiation and the binding of transcription factors [19,20]. Genomic alterations of genes involved in epigenetics, for example, single-nucleotide polymorphisms (SNPs) or copy number variants (CNVs), can lead to epigenetic dysregulation and, ultimately, ASD (Figure 1).

There are over 600 confirmed human genes related to NDD (such as ID and ASD), including DNA methyltransferase 3A (DNMT3A), HECT, UBA, WWE domain-containing E3 ubiquitin-protein ligase 1 (HUWE1), and chromodomain helicase DNA-binding protein 8 (CHD8) [21,22,23]. Emerging evidence suggests that there is a fundamental genetic interference risk in neurodevelopmental and neuropsychiatric disorders [24,25,26]. Many of these genes are essential to the molecular pathways involved in epigenetic regulation, whereas others are encoded to proteins involved in neuronal and synaptic pathways [23,26,27]. However, there is no single underlying cause of ASD or its developmentally related challenges. For a better understanding of the multifactorial aetiologies of ASD, extra work is needed to elaborate on the natural history of its molecular basis and its regulation during significant periods of human development. Apparently, the interaction between genetic and environmental risk factors (GxE) in humans is crucial and is likely facilitated by vital mechanisms such as epigenetics. Thus, the role of genetics, epigenetics, and the environment in ASD risk factors, as well as the collective interaction between them, will require further investigation in order to better classify and diagnose the disorder.

## 2. Body of the Paper

Currently, ASD is considered to have a strong genetic component, likely resulting from interactions between various genes. Previous and current twin studies have reported an estimate of more than 30 percent heritability [14,28,29]. Due to the use of modern technology, such as next-generation sequencing (NGS), many key points of genetic variability in patients with ASD (as compared to the general population) have emerged. Sequence analysis methods, such as whole-genome sequencing (WGS), have many advantages, and they have been used to discover ASD-relevant mutations in individuals within affected families [30,31]. One study on families of four (parents and two affected siblings) observed similar mutations in 31 percent of siblings compared to ASD patients. Thus, such studies underline the genetic heterogeneity of the disorder even within families [30]. However, for all the advantages of genomic sequencing, genetic variant classification still poses an important challenge. More than 200 ASD-associated genes have been specified in various studies, and risk variants have only been recorded in 25–40 percent of cases [32,33,34,35,36]. Nevertheless, in only one percent of ASD cases can a single genetic mutation or copy number variant be correlated, and, thus, the ASD phenotype is unpredictably impenetrable. According to recent studies, more than one thousand autism genes have been investigated. Using the SFARI gene platform, approximately 212 genes (environment interacting genes) have been studied [37,38]. More recently, studies reported that dysregulated gene expression is associated with inflammatory cytokines and behavioural severity in ASD [39,40]. Overall, various genetic variants and six risk-located loci (1q21.1, 3q29, 7q11.23, 16p11.2, 15q11.2–13, 22q11.2) are recognised as being linked with ASD [41,42,43].

Recent genomic studies have identified many common and rare inherited variants in ASD families [44,45]. In autism, the majority of the genetic alteration tracked by heritability is accounted for by common genetic variation, as quantified by SNP-based heritability which is estimated to be around 50 percent [45]. As with other common neuropsychiatric conditions, common genetic variants contributing to autism have small effect sizes, requiring large population studies for identification. The latest and largest published ASD genome-wide association study (GWAS) reported five genome-wide significant loci in an analysis of 18,381 ASD patients [44]. Larger sample sizes will be necessary to identify further loci, which are expected to be found given the substantial estimate of SNP-based heritability [45,46].

Since 2011, a large number of studies have already been published using WGS in ASD populations, with the identification of rare variants in autism susceptibility genes already described or newly identified [30,47,48,49]. These findings have led to an exponential increase in the number of genes potentially related to autism. However, the increase in de novo events in individuals with ASD is widely described in the literature [50], and several studies highlighted the importance of de novo mutations in a situation of family mutational burden due to the presence of common and rare variants inherited from parents [46,50,51].

Several studies have shown that various ASD genomic risk variants are involved in congregating pathways relevant to the natural basis of ASD. These include cell proliferation and differentiation, neural development, synaptic activity, transcriptional regulators, and chromatin modifiers [41,51,52,53]. Interestingly, many ASD-associated genes are expressed in brain tissues during embryogenesis and formation. These functional groups relate to the neurocognitive phenotype of ASD and can improve our understanding of its molecular mechanisms [25,53]. On the other hand, studies on ASD risk-associated genes initiated by genetic mutation may also help in this attempt at comprehension; their findings suggest that some ASD-related genes can increase the risk factor for other neurological conditions, including schizophrenia, motor impairment, epilepsy, sleep disturbance, ADHD, and ID [25,42,54].

In addition, strong evidence supports the role of epigenetics in the molecular aetiology of ASD, some of which indicates that other genetic syndromes may have direct control over its expression. Studies have proven that they contribute to syndromic ASD in approximately 15 percent of cases [55,56]. Functionally, some epigenetic profiles have been reported to be associated with an increased risk of ASD: for example, histone deacetylases (HDACs); lysine demethylases; proteins containing bromo-, chromo-, or Tudor domains; DNA methyl transferases (DNMTs); histone methyltransferases; acetyltransferases; and chromatin remodelling factors. While the indirect effects of epigenes have also been observed in noncoding RNA (ncRNA), the processing and recruitment of methyl-CpG-binding proteins (MBDs) have also been observed, and these are critical in histone modification and transcription regulation [57,58].

Other lines of evidence implicate other epigenetic markers in the expression of ASD, such as differentially methylated variants (DMVs) at specific CpG sites (CpGs) or differentially methylated regions (DMRs). Studies have identified multiple CpGs in a variety of tissue types, including whole blood [59], post-mortem brain tissue [60,61,62], ectodermal cells [63], and lymphoblastoid cell lines (19), as well as sperm from the fathers of children with ASD [64]. However, in concordance with studies on blood–brain DNA methylation, these high correlations have not been frequently observed at specific sites across tissues, which is likely due to genetic influences [65,66]. Several studies have reported DNAm changes in the promoter region in different tissues taken from ASD samples, which has helped identify candidate genes, including methyl-CpG binding protein 2 (MECP2), reelin (RELN), glutamic acid decarboxylase 65 (GAD65), oxytocin receptor (OXTR), ubiquitin-protein ligase E3A (UBE3A), and SHANK3 [67,68,69,70,71,72]. The effect of changes in DMV at specific promoter CpG regions has been found to be modest (approximately twofold) in targeted genes, with a significant gain of methylation (GOM) overall and in a sex-specific manner. Other studies on DNAm have demonstrated the replication of differentially methylated positions (DMPs), which are hypomethylated in the 3′ untranslated region (3′UTR) of genes in the brain samples of ASD individuals, including chromosome 11 open reading frame 21 (C11orf21), proline-rich transmembrane protein 1 (PRRT1), and tetraspanin 32 (TSPAN32) [61,62]. Other results emphasise the presence of functionally relevant genes, such as phosphatase and tensin homolog (PTEN) [73], AT-rich interaction domain 1B (ARID1B) [74], N-methyl-D-aspartate (NMDA) [75], glutamate ionotropic receptor NMDA type subunit 2B (GRIN2B) [76], neurexin 1 (NRXN1) [64], and PRRT1 [61], which have critical roles in physiological function and other molecular pathways [77].

Previous studies have reported the enrichment of genome-wide results by primarily using quantitative trait loci (QTLs) for the purposes of gene expression; supposing these polymorphisms present some ASD risk through regulatory mechanisms, QTLs offer insights into the functional biology of GWAS variants [78,79,80]. Besides investigating the enrichment of polymorphisms that control epigenetic markers (for example, DNAm), understanding the regulatory effects of the ASD epigenome will clearly answer whether the disease’s genetic risks act, in part, through epigenetic regulation. Moreover, the presence of DNAm, detected using SNP profiling or methylation QTLs (meQTLs), has been reported in both autism-associated GWAS and cases of schizophrenia and bipolar disorder, which may indicate genetic overlap with ASD [78,79,81,82,83].

Genetic expression levels and epigenetic markers are molecular sensors for many environmental factors, including diet and chemical toxins, over the course of human development [84]. At critical times throughout development, typical cell programming can be dysregulated by environmental exposures, either exogenous or endogenous, leading to unfavourable long-term health outcomes [85]. Accumulated evidence from observational studies has demonstrated the association of endogenous environmental factors with ASD risks, for example, paternal and maternal age [86,87,88]. Combining these factors, along with genetic and epigenetic findings, has helped in identifying several de novo mutations in such disorders, and the majority of cases have been found to be related to paternal age [33,35]. Furthermore, modest increases in ASD and ADHD risk have been found to be associated with maternal prenatal stress [89,90]. However, while OXTR methylation outcomes were predicted in many studies, nevertheless, correlations between maternal stress and autistic traits have not been observed in genetic and methylation changes in the gene [91,92,93]. Prenatal maternal nutrition, particularly with respect to the folate compound, has been identified as reducing the risk of ASD through its critical role in one-carbon metabolism (OCM) and methionine cycles [94,95,96,97]. At the molecular level, the recycling of homocysteine generates amino acids (cysteine and methionine), which are essential in the process of methylation and antioxidative capacity. This biological process accelerates the formation of S-adenosyl methionine (SAM), supporting epigenetic mechanisms that are important for neurobehavioral development and preventing ASD via nutritional supplements [98]. Still, the molecular mechanisms by which these elements reduce ASD risk have not yet been fully illustrated.

Moreover, exogenous exposures, for example, smoking, medication (valproic acid, selective serotonin reuptake inhibitors), alcohol, zinc deficiency, metal ion toxicity, viral infection, and chemical agents (pesticides, metals, bisphenol A), are often associated with adverse foetal neurodevelopmental outcomes [74,99]. However, the alcohol and smoking risk factors are irrelevant with respect to ASD studies, which could be related to differences in exposure conditions between mother and foetus [100]. In addition, zinc is known to be an essential element in foetal growth and development during pregnancy, as well as in the development of children in general [101,102]. Thus, an extended deficiency of zinc in pregnant women might lead to dysfunction in embryonic growth and development, as well as in synaptic systems. Several reports have demonstrated that the absence of zinc prevents the formation of scaffold structures in the ProSAP/Shank family of proteins, which are the key regulator molecules in synapses [103]. A transgenic study of Shank3+/- and Shank-/- deficient mice using a prenatal zinc-deficient autism animal model indicated diverse brain region abnormalities in different models of ASD [104].

Finally, interactions between the genome and epigenome will allow scientists to enhance methylation studies through increased genomic coverage, expanding our knowledge of the role of ASD-specific biomarkers. For instance, changes in specific epigenetic markers (CpG methylation) in noncoding regions, including promoters, enhancers, silencers, insulators, intergenic regions, and ncRNAs, have been observed during neurogenesis [105,106,107,108,109,110]. Most importantly, alternative types of methylation (non-CpG methylation (CpH, where H = A, C, or T) and 5-hydroxymethylcytosine (5-hmC)) have been reported in the pathogenesis of ASD. The nuclear DNA 5-hydroxymethylcytosine, a base molecule involved in the processing of oxidative demethylation, is highly expressed in brain tissue relative to 5-methylcytosine (5mC) and may reveal significant brain-region-specific regulatory epigenetic signals [111]. Therefore, based on various factors, tackling the issue of heterogeneity in ASD-relative risks by studying more homogeneous subsets of individuals will become an evolving method for better explaining these mechanisms.

In summary, the studies cited in this paper should guide the study of ASD by classifying genetic expressions, epigenetic biomarkers, and environmental conditions for the better prediction of individuals with ASD. Ultimately, they have brought to light several important factors that urgently need to be investigated in order to improve both research design and the interpretation of data going forward.

## 3. Conclusions

Molecular mechanisms can help fill the gap in defining ASD pathogenesis where epigenetic or genetic information alone is inadequate to describe the aetiology of all cases. In terms of effect, genetic and epigenetic profiles are expected to be heterogeneous. Combining genetic, epigenetic, and environmental data is likely to represent a more comprehensive understanding of molecular settings with respect to the aetiology of ASD. The early detection of the molecular basis (genotype, epigenotype) and biological associations with intellectual or ASD-related risk factors will allow health providers to better classify affected individuals, facilitate earlier diagnosis, and improve prognosis. Altogether, improving our molecular understanding of the disorder will also aid in uncovering more homogeneous subgroups of individuals, which will allow for better patient stratification for behavioural and pharmacological therapies.

## Data Availability

All data pertinent to this study are included herein.
